# Identifying differentially regulated subnetworks from phosphoproteomic data

**DOI:** 10.1186/1471-2105-11-351

**Published:** 2010-06-28

**Authors:** Martin Klammer, Klaus Godl, Andreas Tebbe, Christoph Schaab

**Affiliations:** 1 KINAXO Biotechnologies GmbH, Am Klopferspitz 19a, 82152 Martinsried, Germany; 2 Max Planck Institute of Biochemistry, Am Klopferspitz 18, 82152 Martinsried, Germany

## Abstract

**Background:**

Various high throughput methods are available for detecting regulations at the level of transcription, translation or posttranslation (e.g. phosphorylation). Integrating these data with protein networks should make it possible to identify subnetworks that are significantly regulated. Furthermore, such integration can support identification of regulated entities from often noisy high throughput data. In particular, processing mass spectrometry-based phosphoproteomic data in this manner may expose signal transduction pathways and, in the case of experiments with drug-treated cells, reveal the drug's mode of action.

**Results:**

Here, we introduce SubExtractor, an algorithm that combines phosphoproteomic data with protein network information from STRING to identify differentially regulated subnetworks and individual proteins. The method is based on a Bayesian probabilistic model combined with a genetic algorithm and rigorous significance testing. The Bayesian model accounts for information about both differential regulation and network topology. The method was tested with artificial data and subsequently applied to a comprehensive phosphoproteomics study investigating the mode of action of sorafenib, a small molecule kinase inhibitor.

**Conclusions:**

SubExtractor reliably identifies differentially regulated subnetworks from phosphoproteomic data by integrating protein networks. The method can also be applied to gene or protein expression data.

## Background

Protein phosphorylation is one of the most important posttranslational modifications in a living cell. Virtually all cellular processes are regulated by the interplay of protein kinases (proteins that phosphorylate their substrates) and phosphatases (proteins that dephosphorylate their substrates). Phosphorylation events are particularly important in signal transduction, where signals caused by external stimuli are transmitted from the cell membrane to the nucleus. Here, phosphorylation events often act as switches to activate or deactivate their substrate proteins. In many cases, substrates of this process are again kinases. This leads to the signal being propagated along a signalling cascade until it finally triggers a response (e.g. transcription or translation). Although signal transduction pathways are often depicted as a linear series of steps, they may be considerably more complex in reality: many run in parallel, are interconnected and have feedback loops. Aberrations in these cascades can lead to diseases, including cancer [1,2].

To identify phosphorylation sites (phosphosites) on a large scale, mass spectrometry (MS) has become an increasingly important technology [3]. Quantitative MS in particular not only enables detection of phosphosites, but can also measure their relative abundance. By comparing phosphorylation patterns before and after treatment of cells with a drug that interferes with cell signalling (e.g. kinase inhibitors), one can deduce the drug's effect on a signal transduction pathway. Unravelling a drug's mode of action is vital during drug discovery and development, helping to identify new medical applications, suggesting its use in combinational therapy, and predicting the responsiveness of patients [4-6].

Similarly, other global quantification technologies such as microarray and MS-based proteomics can measure the expression of thousands to tens of thousands of genes and proteins, respectively. Often, a few thousand of them are identified as being significantly differentially regulated, but interpreting these results at a single gene or protein level is a tedious and frequently unsuccessful task. However, by integrating these data with protein-protein interaction networks, it is possible to identify significantly regulated subnetworks that can be interpreted directly in a biological context. Moreover, identifying regulated entities from often noisy high throughput data should be supported by this kind of integration.

One simple approach for detecting regulated subnetworks could involve distinguishing between significantly regulated and non-regulated phosphosites by applying standard hypothesis testing procedures such as *t*-statistics or SAM [7] to each phosphosite (the number of data points corresponds to the number of experimental replicates). To avoid too many false positives, one must further apply concepts such as the family-wise error rate (FWER [8]) or the false discovery rate (FDR [9]) for multiple hypothesis testing correction. Subsequently, the resulting list of statistically significant entities can be mapped on pathways or protein-protein interaction networks, and connected subnetworks can be determined. While this procedure may point to regulated subnetworks, it is not an integrated solution, since the significance of each protein solely depends on the data of its own phosphosites, regardless of its interactions with other proteins. More sophisticated approaches use statistic-based techniques to score subnetworks. In these cases proteins are first mapped onto a protein interaction network, and subsequently high-scoring subnetworks are extracted. Ideker *et al*. [10] use an aggregated *z*-score of the form

where *k *is the number of nodes in the subnetwork and *z*_*i *_is the *z *-score of a single protein in the subnetwork *S*. High-scoring subnetworks are then found with a simulated annealing approach [11]. Chuang *et al*. [12] presented a method based on the same idea, but with a greedy search algorithm that specifies a seed and adds the best nodes in the neighbourhood until the aggregated score no longer improves. Subsequently, the significance of the resulting subnetworks is assessed based on null distributions estimated from permuted networks. However, neither method accounts for the network topology, i.e. the degree of interconnections between nodes.

Subsequently, Sanguinetti *et al*. [13] introduced a Bayesian probabilistic model that integrates *a priori *network topology information into the analysis of high throughput data. The authors used Gibbs sampling [14] to obtain suitable posterior probabilities and thus derived subnetworks. A major drawback of this method, however, is the missing significance assessment for the resulting subnetworks.

All methods described above used either only a subset of known protein-protein interactions or KEGG pathways [15] for their assessment. To obtain the most information from such investigations, and considering that canonical pathway databases like KEGG are rather static and contain only a limited number of interactions, it seems natural to use larger and frequently updated protein-protein interaction network databases such as STRING [16] or FunCoup [17].

Here, we introduce a Bayesian probabilistic model that combines local as well as topological information, i.e. information about regulation of a certain node and information about the connectivity with its neighbours. Identification of subnetworks is carried out using a genetic algorithm (GA [18]), followed by performing a significance analysis based on a global rank test [19]. As a special feature, the significance test not only considers subnetworks, but also single nodes that are not part of any larger subnetwork. This makes the proposed method a powerful tool to uncover both differentially regulated subnetworks and differentially regulated single proteins. The performance was assessed on an artificial data set as well as on a comprehensive phosphoproteomics data set [20].

## Methods

### Data pre-processing and z-score calculation

The input of the proposed method is formed by a table with *n *rows and *m *columns; *n *being the number of detected phosphosites and *m *the number of biological replicates (i.e. MS measurements of experiments using identical settings but conducted independently). Several replicates (at least 3-5) are necessary to reliably identify differential phosphorylations. Each value in this table represents a ratio between the degree of phosphorylation under two conditions (e.g. the extend of phosphorylation of a specific site in cells treated with a drug versus its degree in untreated cells).

Log-transformation is preferred before calculating the *z*-score, since the distribution of the transformed ratios is closer to normal. Subsequently, the log-ratios *x*_*ij *_of phosphosites *i *= 1, ..., *n *and replicates *j *= 1, ..., *m *are further transformed to *z*-scores (referred to as single *z*-scores) using the formula:(1)

where *μ*_0 _= 0, since it is expected that the majority of phosphosites are not differentially regulated and therefore their log-ratios are 0, and  the standard deviation across replicates estimated on the entire data set. Further, a combined *z*-score for each phosphosite over all replicates is calculated as:(2)

Not all phosphosites are detected in every experimental replicate. The resulting missing values are simply ignored, so, for example, if three replicates have been conducted and a given phosphosite was only detected in two of them, *m *is set to 2 for this site and the combined score is calculated based on the two available *z*-scores.

### Protein network preparation

In this work STRING [16] was chosen as the source for protein-protein interactions. STRING is a comprehensive resource that combines a vast number of databases derived in different ways (e.g. experimentally determined interactions, gene neighbourhood data, or data acquired via text mining) and is able to transfer homology information across organisms. Obviously the method presented here is not limited to STRING and can also be used in combination with other protein-protein-interaction databases. Depending on the context of the study databases like HomoMINT [21], HPRD [22], or FunCoup [17] may be preferable.

In STRING, all interactions are assigned with a confidence value ranging from 0 to 1. To retain only high confidence interactions, a very conservative cut-off value of 0.995 is used. While this cut-off may seem too high, there is a valid reason for it: some interactions reach very high confidence values (> 0.99), although the evidence is only from text mining, which was considered too weak evidence. Furthermore, analysis of canonical pathways showed that virtually all known interactions pass this high cut-off of 0.995. Applying this cut-off, an interaction network of approximately 10,000 interactions between 2,997 proteins is obtained (STRING version 8.1).

**Figure 1:**
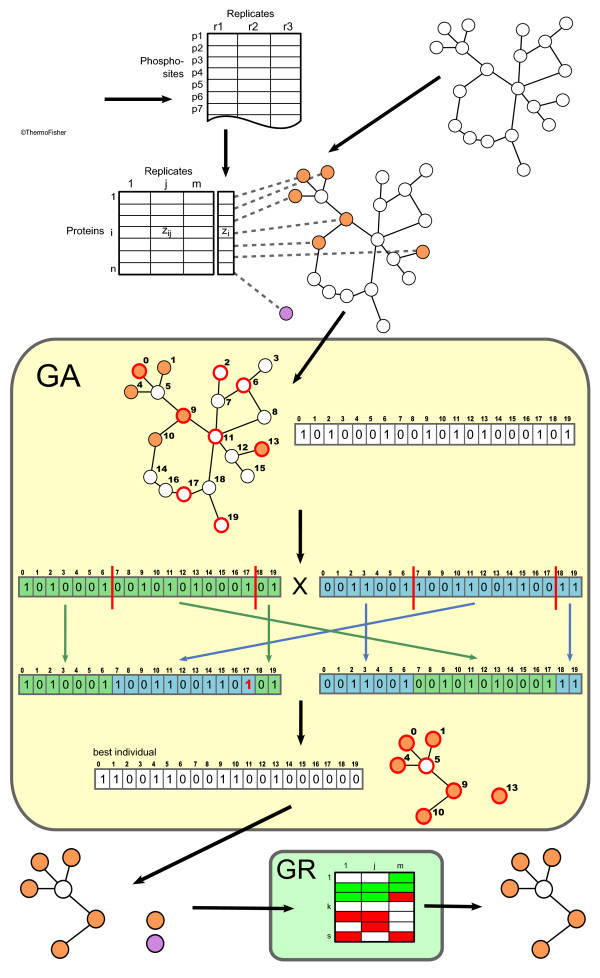
**Workflow of the subnetwork extraction**. First, single and combined *z*-scores are calculated from the phosphoproteomics data set and subsequently mapped on an interaction network (orange nodes). Proteins that do not occur in the interaction network are stored in a separate list (violet node). For the genetic algorithm (GA) procedure the network is encoded into a binary vector, where 1 codes for the associated node being active (i.e. part of a regulated subnetwork) and 0 inactive. The GA runs for a defined number of generations (exemplarily, the two-point crossover step in combination with a single-point mutation is depicted), and the strongest individual of the final generation encodes for the globally best achievable solution (here, this would be a subnetwork containing six nodes and a single-node network). Finally, the global rank (GR) significance test is performed on both extracted subnetworks and single nodes (or-more generally-single-node subnetworks) resulting in a set of significantly regulated subnetworks (only one in the depicted example).

Subsequently, the phosphoproteomic data is mapped on the network (see upper part of Figure 1). Before doing so, the list of phosphosites has to be aggregated to a list of proteins, with one *z*-score per protein and replicate. This is done by simply assigning the values of the phosphosite with the highest combined *z*-score among all phosphosites of a protein to this protein. Then, each protein is mapped on the interaction network, where each node has *m *single *z*-scores and the combined *z*-score. Nodes that do not have a corresponding entry in the phosphoproteomics data set are thought of being not regulated and thus their *z*-scores are set to 0. On the other hand, proteins on the list that do not occur in the network are added but without any connections in order to give them the chance of being identified as regulated single proteins later on. In the genetic algorithm described below, only nodes in the interaction network will be considered; the set of unconnected nodes will be used again when it comes to significance assessment in the final step of the method.

### Bayesian probabilistic model

A probabilistic model that takes into account the above derived *z*-scores and the network topology was developed. Let *c*_*i *_∈ {0,1} be the latent class variable, with *c*_*i *_= 1 if node *i *belongs to a differentially regulated subnetwork and *c*_*i *_= 0 if not. Note that the approach can easily be generalized to three classes, if up- and down-regulated subnetworks shall be distinguished. Given the combined *z*-scores *z*_1_, ..., *z*_*n *_derived from the observations, the posterior probability of the subnetwork configuration (*c*_1_, ..., *c*_*n*_) is(3)

where the right-hand side is obtained by applying Bayes' theorem. The denominator *p *(*z*_1_, ..., *z*_*n*_) does not depend on the *c*_*i *_and can be ignored when maximizing the posterior probability. Since the observed data of node *i *are mutually conditionally independent (given the other nodes' class variables) and depend only on the class variable of the node itself, the conditional probability can be written as(4)

Normal distributions (*μ*, *σ*) with *μ *= 0 and *σ *= 1 or *σ *= *σ *_*z *_are assumed:(5)

The prior probability for the subnetwork configuration *p *(*c*_1_, ..., *c*_*n*_) is derived analogously to the derivation of the joint probability distribution from conditional probabilities in Bayesian networks. Let *N*_*i *_be the set of parents of node *i*. If the protein interaction network was a directed acyclic graph and the joint distribution fulfilled the Markov condition, the following equality would hold [23]:(6)

Clearly, protein-protein interaction networks are no directed acyclic graphs. Nevertheless, the prior can be modelled by applying this theorem, if *N*_*i *_is now defined as the set of neighbours of node *i*. The conditional probabilities are modelled similarly to [13]:(7)

and(8)

or equivalently(9)

where the parameter *α *determines the weight of the network structure, and |*N_i_| *is the number of neighbours. For very large *α*, the posterior probability is not influenced by the network structure. Taking the logarithm of Equation (3), inserting above equations, and ignoring the constant summands, the log posterior probability is:(10)

The model parameters *α *and *σ*_*z *_are fixed. In principle, they could be handled as unknown parameters in the Bayesian model, with the effect that the joint posterior probability would have to be maximized for (*c*_1_, ..., *c*_*n*_), *α *and *σ*_*z*_. Since the results turned out to be rather insensitive to variations in *α *and *σ*_*z *_(see *Results and Discussion*), the model and the optimization were simplified by *a priori *fixing of these parameters.

### Subnetwork extraction

To maximize the posterior probability, the optimal combination of the nodes' class associations (i.e. whether a protein is part of a regulated subnetwork to be extracted or not) has to be found. Since this problem is NP-hard [10], a heuristic strategy has to be applied. Genetic algorithms (GAs) are particularly well-suited for this kind of binary-valued combinatorial problem, since they are able to find close-to-optimum solutions even in complex scoring landscapes with many local optima (see e.g. [18] for more details). An overview of a standard GA workflow can be found in Additional file 1.

To apply a GA to the subnetwork extraction problem, the network has to be encoded into a vector (i.e. an individual's chromosome). Here each node in the network was assigned a consecutive index value that represents the position of this node in the vector. The values in the vector are binary: 1 meaning that the corresponding node is part of a regulated subnetwork, and 0 that it is not (see also Figure 1). Initially, values of these binary vectors are randomly generated, one for each of the 1000 individuals used. According to the Bayesian scoring function described above, the fitness of each individual is evaluated and 100 individuals are selected and used for breeding. Selection of these individuals is performed using the tournament selector (cf. [24]), which randomly draws a subset of individuals and then determines the fittest within this subset. By repeating these steps 100 times, the 100 parent individuals are selected. Tournament selection ensures that average-performing individuals also have some chance to reproduce, which reduces the risk of premature convergence. Recombination of the selected individuals is carried out with two-point crossover, that is, the chromosomes of two parents are cut at two identical, random points *c*1 and *c*2, and the genes in the range [*c*1, *c*2] are crossed (see also Figure 1). Mutation, which is a simple bit ip, occurs with a probability of 0.05. The newly created offspring's fitness is assessed, and the fittest offspring replaces the weakest individual in the parental generation. Then the algorithm continues with the selection of a new set of parents. The algorithm is run for 5000 generations, an empirically determined value, from where on no more appreciable improvement is observed. The best solution (represented by the individual with the highest fitness value in the final generation) is then used to extract all subnetworks from the entire network by starting at a given node, checking all neighbours for their class association, and iteratively adding all neighbours that belong to a regulated subnetwork. To avoid cycles, every node is flagged after it has been checked, and if no more neighbours are to be added to the current subnetwork in a certain iteration step, another as yet unchecked node is used as the starting point for the next subnetwork. This is repeated until no unchecked nodes are left, and therefore all subnetworks are detected. The *z*-score of a subnetwork is then defined as:(11)

where *z*_*i *_is the combined *z *-score of a protein as described in (2), *S*_*s *_is the set of proteins in the subnetwork, and |*S*_*s*_| is its size. The absolute value of *z*_*i *_is taken, since it is not know *a priori *whether the interaction between two proteins is activating or inhibiting, and therefore this distinction is not made. Rather only the degree of regulation is taken into account. When analysing gene or protein expression data, however, the direction of regulation may be important and should not be ignored. In such cases, the signed values can be used. In some cases, a subnetwork may contain only one node, which is not an issue, since both significant subnetworks and single nodes shall be determined anyway.

### Significance evaluation

Once regulated subnetworks are extracted, one has to determine their statistical significance. Single nodes (those that could not be mapped on the network but had been detected in the phosphoproteomics experiment) are regarded as subnetworks with only one member and are thus added to the list of subnetworks. The significance test is based on a modified version of the global rank test [19].

The main idea of this method is to identify differentially regulated entities (genes, proteins or subnetworks) not based on hypothesis tests conducted for each entity independently, but rather based on the entire set of entities at once. Under the null hypothesis that entities are neither up- or down-regulated, the authors state the theorem of random ordering, i.e. that no entity can rank consistently high or low across all replicates. On the contrary, those entities that do consistently rank top or bottom in all replicates are identified as being significantly regulated. The number of identified significant entities will then solely depend on the number that determines how many entities are considered *top *or *bottom *ranked (here denoted as *N*), e.g. if *N *is chosen to be a small number, only a few entities or none at all will be among the *top-N *or *bottom-N *across all replicates.

Raising *N *not only increases the number of identified significant entities, but also the expected number of false positives. As described in [19], this number of false positives can be estimated non-parametrically from the empirical null distribution. The idea for this procedure is that a non-regulated entity has the same probability of ranking *top-N *as ranking *bottom-N*. In other words, under the null hypothesis an entity has the same probability of ranking *top-N *across all replicates (denoted as TTT for three replicates [*R *= 3]) as ranking *bottom-N *across all of them (BBB) or *top-N *in the first two and *bottom-N *in the third (TTB). The same is true for all 2^*R *^= 8 classes of possible combinations of high and low ranks. Entities in the TTT and BBB classes are differentially regulated, and those in the remaining 2^*R *^- 2 = 6 classes are not. By dividing the average number of entities in the 6 non-consistently regulated classes by the number of those in one of the regulated classes, for each *N *the FDR can be estimated (once for up- and once for down-regulated entities). Different values of *N *can now be tried until the desired FDR level is reached (cf. algorithm in Table 1, line 10 - 19).

**Table 1:** Overview of significance evaluation

1: *A *= *z*-transformed phosphoproteomic data (*n *phosphosites, *m *replicates)
2: *STRING *= STRING interaction data
3: *origSN *= list of extract subnetworks from *STRING *using *A*
4: *flippedSNs *= container for flipped subnetwork lists
5: **for all **s ∈ Cartesian product {-1, +1}^*m *^without {(-1,...,-1), (+1, ..., +1)} **do**
6: *flippedA *= multiply values in column (1, ..., *i*, ..., *m*) of *A *with the value at index *i *in *s*
7: add list of extracted subnetworks from *STRING *using *flippedA *to *flippedSNs*
8: **end for**
9: *FDR *= 1.0
10: *N *= *n*
11: **while ***FDR *> desired FDR cutoff **and ***N >*0 **do**
12: *origCount *= count subnetworks that are among the *N *most-regulated ones across all replicates in *origSN*
13: *flippedCount *= 0
14: **for all **flipped lists of subnetworks **in ***flippedSNs ***do**
15: *flippedCount *= *flippedCount *+ number of subnetworks from list of flipped subnetworks that are among the *N *most-regulated ones across all replicates
16: **end for**
17: *FDR *= (*flippedCount*/number of lists in *flippedSNs*)/*origCount*
18: *N *= *N *- 1
19: **end while**
20: **if ***N *> 0 **then**
21: **return **list of subnetworks that are among the *N *+ 1 most-regulated ones across all replicates in *origSN*
22: **else**
23: **return **empty list
24: **end if**

The algorithm for significance evaluation in pseudocode.

For the application to subnetworks the method estimating false positives has to be modified, since the subnetworks' *z*-scores have non-negative values only, which means that *bottom-N *ranking subnetworks would be the ones with the weakest regulation. To overcome this problem, one first has to introduce another way of counting entities that fall under the non-consistently regulated classes, since the bottom ranked no longer represent differentially regulated entities. In this new counting process, not simply the entities in the non-regulated classes are counted but rather the signs of the replicates' *z*-scores are alternately changed (cf. algorithm in Table 1, line 5 - 8) and subsequently the number of entities that consistently rank top across all replicates after this transformation are counted (cf. algorithm in Table 1, line 14 - 16). In the case of the TTB class, for example, rather than determining the number of entities ranking *top-N *in the first two replicates and *bottom-N *in the third, the signs of the third replicate's *z*-scores are flipped and one determines the number of entities now ranking *top-N *across all three replicates (those that are now in the TTT class). Note that both counting methods yield the same results, since it makes no difference whether one counts the number of *bottom-N *entities of a given replicate or the number of sign-flipped *top-N *ones.

The *z*-score of a subnetwork is as defined in (11), where *z*_*i *_is the combined score over all replicates. To find subnetworks that are top ranked across all replicates *z*-scores have to be calculated for each replicate separately:(12)

where *z*_*ij *_is calculated with equation (1). The problem here is that two nodes within a subnetwork - one with a highly positive and one with a highly negative score - would mutually neutralize each other. This effect is undesirable, since the direction of regulation does not matter for the application described here. On the other hand, if the absolute value of *z*_*ij *_was taken, the sign-flipping used to calculate the FDR would have no effect. Thus, a trick is applied: if the sign of a given *z*_*ij *_is in accordance with the *z *-scores of all replicates (i.e. if it has the same sign as ∑_*j' *_*z*_*ij'*_), *z*_*ij *_will contribute positively to the score , if not it will contribute negatively:(13)

where sgn is the sign function. This equation is applied in line 12, 15 and 21 of the algorithm in Table 1 to find consistently top ranked subnetworks.

Entities that lack data in one replicate are accepted as differentially regulated, if they rank top in the remaining *m *- 1 replicates. This criterion compensates for missing data, a particular problem in mass spectrometry experiments.

### Implementation

Pre-processing, *z*-score calculation and generation of the artificial data set was performed using Matlab. The SubExtractor algorithm is written in Java using the GA library Jenes (http://jenes.ciselab.org; version 1.2.0) and made available for download online at http://www.kinaxo.de/SubExtractor. Java version 5.0 or higher is required to run the program. Network diagrams were created with Cytoscape [25].

## Results and Discussion

### Artificial data

To benchmark and assess the proposed method, the algorithm was tested with artificial data. For this purpose, scale free networks based on the algorithm described in [26] with 1000 nodes and an average connectivity of approximately 3.5 were generated. Artificial *z*-scores were produced by sampling values for 969 nodes from a normal distribution with *μ *= 0 and *σ *= 1 representing non-regulated proteins (background distribution); three times for each entity to simulate experimental replicates. The values for the 31 regulated nodes were determined in a two-step procedure. First, the means *x *were sampled from a normal distribution with *μ *= 0 and *σ *= 5. Second, the actual replicate values were generated by drawing three times from a normal distribution with *μ *= *x *and *σ *= 1. All 31 regulated nodes are connected with each other forming one regulated subnetwork, which should be extracted by the algorithm as accurately as possible. This data generation process was repeated ten times, resulting in ten artificial data sets.

**Figure 2:**
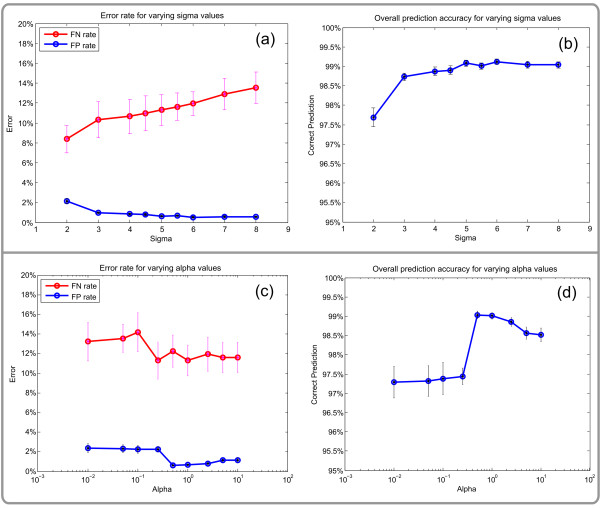
**SubExtracor's performance on artificial data**. Ten artificial data sets were generated to assess the prediction quality of SubExtractor. The top figures (2a and 2b) show the performance for varying *σ*_*z *_values and a fixed *α *of 1.0. The figures at the bottom (2c and 2d) depict the mean accuracy for varying *α *values ranging from 0.01 to 10 and a fixed *σ*_*z *_of 5.0. Nodes sampled with the background distribution (*σ *= 1) are the negatives, those coming from the distribution with *σ *= 5 are the positives. The FN rate is defined as , the FP rate as  The overall prediction accuracy is . Error bars display the standard error of the mean over the ten generated data sets.

Different *σ*_*z *_and *α *values were used to assess the subnetwork reconstruction. Values of the *σ*_*z *_parameter ranged from 2.0 to 8.0. The parameter *α *that determines the weight of the network structure on the entire Bayesian score was varied within a range of 0.01 to 10. Figure 2 shows the mean prediction accuracies over all ten artificial data sets at an FDR level of 0.05 (with 100 GA individuals and 3000 GA generations). Not surprisingly, a *σ*_*z *_value of 5.0 delivers the best results (see Figures 2a and 2b), which is the same value as used for sampling the regulated nodes. At the same time, the graphs show a rather weak dependence on its exact value. Only very small values (e.g. *σ*_*z *_= 2.0) lead to a considerable increase of false positive predictions (see Figure 2a), which was also expected since such values are already very close to the *α *value of the background distribution. For *α*, the best results could be obtained by setting its value between 0.5 and 2.5 (see Figures 2c and 2d). Lower values cause the model to put too much weight on the network structure, which causes especially weakly regulated nodes that are only connected to strongly regulated ones to be spuriously incorporate into the regulated subnetwork. Higher values, on the other hand, result in under-weighting of the network structure, which in turn causes an incorporation of moderately regulated nodes even if the majority of their neighbours are not regulated at all. Furthermore, one can clearly see that the results are not sensitive to the exact values of the parameters *α *and *σ*_*z*_, which supports the decision to fix them *a priori*. However, the overall prediction accuracy steeply increases between *α*-values of 0.25 and 0.5 (see Figure 2d). This is due to the effect that if a non-regulated node has only one connection to a well-regulated node (and no other connections) and *α *is smaller than a critical value *α*_*c*_, it will be added to the differentially regulated subnetwork, just because of this special connectivity property. To avoid this undesired effect, *α *has to be chosen(14)

(the derivation of this formula and further explanation can be found in Additional file 1). For *σ*_*z *_= 5.0 this leads to valid *α *values of *α *> 0.25, which explains the large number of false positives for values ≤ 0.25 (as depicted in Figure 2c).

**Figure 3:**
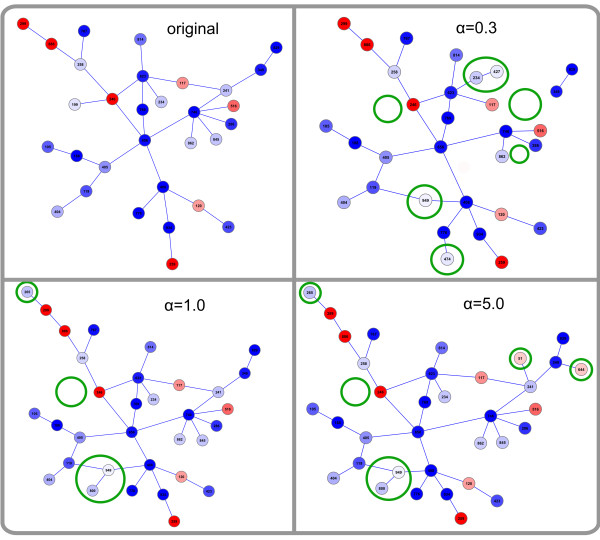
**Example of subnetwork extraction for one artificial data set**. The top left area shows the network of 31 nodes that have been sampled from the normal distribution with *μ *= 0 and *σ *= 5, thus being the regulated ones in the artificial data set containing 1000 nodes in total. The remaining three areas show networks reconstructed by the proposed algorithm using different values of the parameter *α*. The colouring represents the level of regulation, where down-regulated nodes are coloured blue, up-regulated ones red and non-regulated nodes white (the darker the colour the stronger the regulation). The differences between the original and the reconstructed subnetworks are highlighted by green ellipses.

A detailed graphical view of the *α *parameter's impact on the prediction results can be seen in Figure 3, where the originally regulated network and three examples of networks reconstructed by the method (for a fixed *σ*_*z *_of 5.0 and alpha set to 0.3, 1 and 5) are depicted. A small value of *α *just above *α*_*c*_(Figure 3 top right) causes an acquisition of some low regulated nodes (the bright ones within the green circles), since the Bayesian score is mainly influenced by the network structure. On the other hand, one node is lost since it has many connections to non-regulated nodes but only a few to regulated ones (7 and 3, respectively) causing the network to break apart (upper right empty circle). For *α *= 0.3, the algorithm extracts 4 false positive nodes while missing 3 true positives. On the contrary, a high value of *α *= 5 (Figure 3 bottom right) causes the algorithm to almost entirely ignore topology information, and thus nodes are incorporated mostly according to their level of regulation. This leads to false positive classification of 5 nodes, of which 4 are fairly well-regulated (i.e. although they were sampled from the background distribution they received a high score by chance), and the fifth one-although not regulated itself-acts as a link to one of the well-regulated false positives. Only one of the true positives was missed. The results for *α *= 1 (Figure 3 bottom left) form a good compromise between the previous two settings, as neither of the two score components is over-weighted. This reconstructed network has a lower number of false predictions (3 false positives and 1 false negative), which is a very satisfying result given that many nodes classified as regulated show very moderate regulation (weaker than some nodes from the background distribution). To demonstrate the advantage of SubExtractor over a method that does not take network information into account, the original global rank test [19] was applied to the artificial data sets. The average false negative rate of this method at an FDR level of 0.05 was 29.0%, the average false positive rate was 0.2% (the best results of SubExtractor with *α *= 1.0 and *σ*_*z *_= 5.0 were 11.3% and 0.7%, respectively). Although SubExtractor produces slightly more false positives, the superior capability to detect true positives even if they are only moderately regulated is obvious.

### Sorafenib mode of action study

Subsequently the algorithm was applied to a real phosphoproteomics experiment, in which triply SILAC-labeled PC3 cells were incubated with the small molecule kinase inhibitor sorafenib (Nexavar^®^, Bayer HealthCare) for 30 and 90 minutes, including a control [20]. Proteins were extracted and digested, and phosphopeptides were enriched using SCX-IMAC/TiO_2_. High resolution LC-MS/MS data of three biological replicates were processed using MaxQuant [27].

A total of 15, 800 class-1 sites (i.e. highly confident phosphosites) on 3, 900 unique proteins were detected. Since two time points are not sufficient to perform any sensible time-course analysis, the more time point with the more extreme absolute value of its average log ratios (either  or ) over the three replicates is taken for each phosphosite. Phosphorylation sites were then pre-processed as described in the *Methods *section. Interaction data was taken from STRING version 8.1 [16] and pre-processed as described in *Methods*. The *α *parameter was set to 1.0, based on the observations made from artificial data. *σ*_*z *_was estimated by applying the original global rank method [19] to the list of phosphosites and calculating the standard deviation of the resulting differentially regulated sites' combined *z*-scores, which led to a value of *σ*_*z *_= 5.5. Other parameter values were also tested, resulting in very similar networks (data not shown). This supported the findings from the artificial data study, where it has been shown that results are rather insensitive to the exact parameter values.

**Figure 4:**
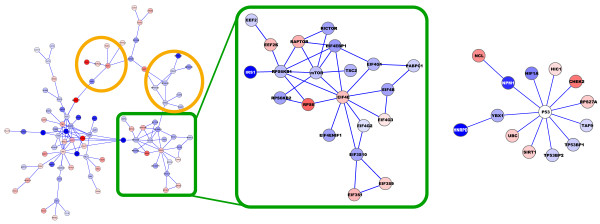
**Subnetwork extraction for sorafenib mode of action study**. The largest two resulting subnetworks are shown (blue nodes are down-regulated, red ones up-regulated) Proteins in the orange circles belong to the MAPK pathway, which is known to be affected by sorafenib. The green rectangle depicts the part of the largest subnetwork that belongs to the mTOR pathway, has not previously been reported to be affected by sorafenib. The network on the right hand side shows important strength of the algorithm, i.e. that subnetworks are also reconstructed if the centre node (i.e. the hub) is not detected to be regulated.

At an FDR level of 0.05, the proposed algorithm was able to reconstruct 21 significantly regulated subnetworks with 168 nodes in total. Additionally, 225 individual proteins were identified as significantly regulated. A selection of the results are depicted in Figure 4. Besides parts of the MAPK pathway, which is known to be affected by sorafenib, the largest network contains a substantial fraction of proteins from the mTOR pathway, which was previously not known to be affected. Subsequent enrichment analyses of the mTOR KEGG pathway confirmed the results of SubExtractor (p-value < 0.005 using Fisher's exact test; data not shown). In particular, a substantial number of translation initiation factors (eIF's) show regulation of phosphorylation upon sorafenib treatment. Further biological interpretation and validation will be published in [20].

Another example in Figure 4 depicts a subnetwork centring the tumour suppressor p53. This example shows the strength of the method to reconstruct networks, even if the hub of the subnetwork is not phosphorylated, not detected, or not regulated. Greedy search methods that grow subnetworks by selecting a seed and iteratively expand it by adding regulated neighbours cannot identify such subnetworks. The complete result in Cytoscape session file format is provided as Additional file 2, and in Excel format as Additional file 3.

### Normal distribution assumption

Both regulated and non-regulated phosphosites were assumed to be normally distributed with different variances (1 and *σ*_*z*_, respectively). Hence, a mixture model of these two distributions should describe the experimental data well. To further investigate this assumption we created a probability plot, which is used to assess whether data comes from a given distribution. However, the plot (see Additional file 1) indicates that a mixture model of standard normal and *t *location scale distribution (essentially a normal distribution with heavier tails) fits the data better than the mixture of the two normals.

Next, the impact of the different distributions on the SubExtractor results was assessed by modelling the regulated data (cf. Equation 5) with a *t *location scale distribution with the mean parameter set to 0, a variance of  and 6 degrees of freedom (estimated based on the fit above). However, the results of the *t*-normal mixture model were strikingly similar to those of the normal-normal mixture, suggesting that the slightly better fit of the former does not increase the prediction accuracy (compare Additional files 2 and 4). Given the simplicity of normal distributions (i.e. in comparison to *t *distributions no degrees of freedom have to be estimated) and the comparable results, the normal-normal mixture model was considered preferable.

### Alternative STRING network preparation

Instead of applying a very conservative cut-off of 0.995 to the combined STRING interaction score, an alternative version was created where the score was re-computed omitting text mining evidence. The computation was performed according to [28], and should avoid very high confidence values that are only due to sometimes doubtable text mining evidences. For the re-computed score the cut-off was set to 0.95, which is still conservative but increases the number of interactions by 80% and the number of involved proteins by 20%. SubExtractor was then run with this version of network information and the sorafenib data (all parameters were left unchanged). While the general tendency of affected pathways and groups of proteins is very similar, the nodes of the largest network have roughly doubled making it rather complex (see Additional file 5). The decision on which network data file to use is left to the user, as it may depend on the application whether he prefers rather complex but comprehensive networks or smaller networks that are easier to interpret. Both files are available for download at http://www.kinaxo.de/SubExtractor.

## Conclusion

Here, we propose a novel method, SubExtractor, for extracting differentially regulated subnetworks from protein-protein interaction networks based on data from global quantification technologies. The core of the method is formed by a Bayesian probabilistic model that accounts for the regulation of proteins as well as for the network structure. A genetic algorithm was implemented to find the subnetworks that maximize the Bayesian score. Furthermore, a global rank significance test was used to distinguish between significantly regulated subnetworks and those formed by chance.

Although some parts of the method have already been presented elsewhere (cf. *Introduction*), the main advantage of the proposed method is the combination of the three main parts: Bayesian probabilistic model, powerful heuristics in the form of GA and rigorous significance testing. To our knowledge, none of the existing methods offer this combination. Additionally, the significances of single nodes (i.e. either proteins that could not be mapped on the interaction network or extracted single-node networks) are also assessed, which makes separate statistics on a protein scope redundant. Using data from the comprehensive STRING database guarantees high reliability of the detected interaction subnetworks. The method was tested with artificial data sets and showed a high level of reconstruction accuracy. Knowledge from this study was transferred to a mode of action study, where SubExtractor revealed differentially regulated subnetworks from known and novel sorafenib-affected pathways, e.g. the MAPK-and mTOR-pathway, respectively. These regulated subnetworks led to creating new hypotheses about the mode of action of sorafenib in prostate cancer PC3 cells [20]. Furthermore, the subnetworks may also play an important role in discovering biomarkers. It has been shown [12] that identified markers for class prediction are more reproducible if their identification is based on subnetworks rather than single genes. Generalization of the proposed method for identifying subnetwork markers used for class prediction will be the focus of future work.

## Authors' contributions

MK designed and implemented the algorithm, performed the statistical analyses and drafted the manuscript. KG and AT designed and supervised the biological experiments and helped with the interpretation of the results. CS assisted in designing the algorithm, participated in drafting the manuscript and supervised the project. All authors read and approved the final manuscript.

## Supplementary Material

Additional file 1 **Supplementary document**. This document contains an introduction to Genetic Algorithms, a guideline for finding the lower bound of parameter *α*, and the probability plot comparing a mixture model of two normal distributions with a mixture of a normal and a *t *location scale distribution. Click here for file

Additional file 2 **Complete set of extracted subnetworks from sorafenib data**. This file contains the set of all significant subnetworks and single nodes that have been extracted from the sorafenib mode of action data with SubExtractor. Two normal distributions as described in the *Methods *section were used to model the distribution of non-regulated and regulated phosphosites. The open source software Cytoscape http://www.cytoscape.org/ is required to view this file. If the file has the format *.zip you have to re-name it to *.cys in order to be able to open it with Cytoscape. Click here for file

Additional file 3 **List of extracted proteins from sorafenib data**. This Excel file contains a list of all proteins that are part of a significantly regulated subnetwork extracted from the sorafenib mode of action data, along with their Uniprot accession numbers, combined *z*-scores and subnetwork affiliation. Click here for file

Additional file 4 **Complete set of extracted subnetworks from sorafenib data using a t distribution**. This file essentially contains the same data as Additional file 2, but this time a *t *location scale distribution as described in the *Normal Assumption *subsection was used to model the distribution of differentially regulated phosphosites. The open source software Cytoscape http://www.cytoscape.org/ is required to view this file. If the file has the format *.zip you have to re-name it to *.cys in order to be able to open it with Cytoscape. Click here for file

Additional file 5 **Complete set of extracted subnetworks from sorafenib data using the alternative STRING data**. This file contains the set of all significant subnetworks and single nodes that have been extracted from the sorafenib mode of action data with SubExtractor using the alternative STRING data described in the *Alternative STRING network preparation *subsection. The open source software Cytoscape http://www.cytoscape.org/ is required to view this file. If the file has the format *.zip you have to re-name it to *.cys in order to be able to open it with Cytoscape. Click here for file
