# Supplementary material for: Identifying differentially regulated subnetworks from phosphoproteomic data
Source: BMC Bioinformatics. 2010 Jun 28;11:351. doi: 10.1186/1471-2105-11-351 (PMC2914729; doi:10.1186/1471-2105-11-351)
Supplement: Additional file 1 — Supplementary document. This document contains an introduction to Genetic Algorithms, a guideline for finding the lower bound of parameter α, and the probability plot comparing a mixture model of two normal distributions with a mixture of a normal and a t location scale distribution. [file 1471-2105-11-351-S1.PDF]

Identification of differentially regulated subnetworks from phosphoproteomic data (Supplements)

Introduction to Genetic Algorithms (GAs)

GAs mimic the process of biological evolution. The primary component of the GA is the individual, which contains exactly one chromosome and one fitness value. A chromosome in GA language is a vector of values (in the simplest way in binary form) representing one distinct solution for the optimization problem. The fitness value determines the quality of the corresponding solution encoded by the chromosome. Depending on the underlying fitness function it is desirable to either maximize or minimize the fitness value.

A typical GA has at least tens or hundreds of different individuals with different chromosomes. As the algorithm evolves, individuals are selected according to their fitness value and bred using crossover and mutation operators to create new offspring and thus new solutions to the problem. Subsequently, some weak individuals (i.e. individuals with low fitness value) from the parental generation are replaced by strong offspring individuals and the process starts over again. According to the building block hypothesis, small areas with superior fitness on different chromosomes are thus iteratively combined into longer ones, leading to a steady increase in fitness (not necessarily for each individual but at the level of the entire population). Random mutations reduce the risk of getting trapped in a local optimum. The general workflow for a GA is depicted in Supplementary figure 1.

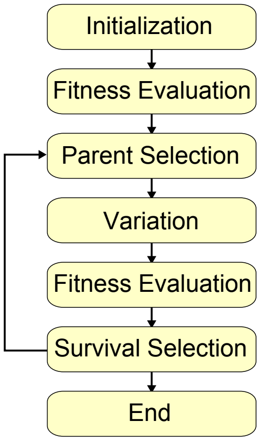

Supplementary figure 1: Schematic GA workflow. First, the individuals' chromosomes are initialized. Then, their fitnesses are evaluated and parents for the first reproduction are selected. Subsequently, variation, i.e. recombination of the parents' chromosomes and mutation, takes place. This is followed by the fitness evaluation of the newly created individuals and subsequent survival selection. In this step low-performing individuals of the parental generation are replaced by high-performing offspring. Steps 3 to 6 are repeated until a certain termination condition (e.g. number of generations or satisfying solution) is fulfilled.

Lower bound for parameter α

As described in the *Artificial data* subsection of the main article, a too small value for α will lead to incorporation of unregulated nodes if their only connection is to a well-regulated one. To avoid this the α value should be chosen such that an unregulated node with only one well-regulated neighbour always gets a higher score when it is flagged as inactive, i.e. not part of an differentially regulated subnetwork. More formally, this requirement can be expressed based on Equation (10) in the main article with the equation

$$\ln\left(\mathcal{N}(0|0,1)\right) + \ln\left(\alpha + 0\right) > \ln\left(\mathcal{N}(0|0,\sigma_z^2)\right) + \ln\left(\alpha + 1\right), \tag{1}$$

which can be rewritten as

$$\alpha > \alpha_c = \frac{1}{e^{\Delta} - 1}, \tag{2}$$

where

$$\Delta = \ln \frac{\mathcal{N}(0|0,1)}{\mathcal{N}(0|0,\sigma_z^2)}. \tag{3}$$

By substituting Δ in (2) by (3) one gets

$$\alpha_c = \frac{1}{\frac{\mathcal{N}(0|0,1)}{\mathcal{N}(0|0,\sigma_z^2)} - 1} \tag{4}$$

or equivalently

$$\alpha_c = \frac{\mathcal{N}(0|0,\sigma_z^2)}{\mathcal{N}(0|0,1) - \mathcal{N}(0|0,\sigma_z^2)}. \tag{5}$$

Equation (5) is then used to calculate the lower bound of reasonable values for α. Some examples for varying σ_z values are:

$\sigma_z = 3$: $\alpha_c = 0.5$
$\sigma_z = 5$: $\alpha_c = 0.25$
$\sigma_z = 10$: $\alpha_c = 0.11$

Probability plot

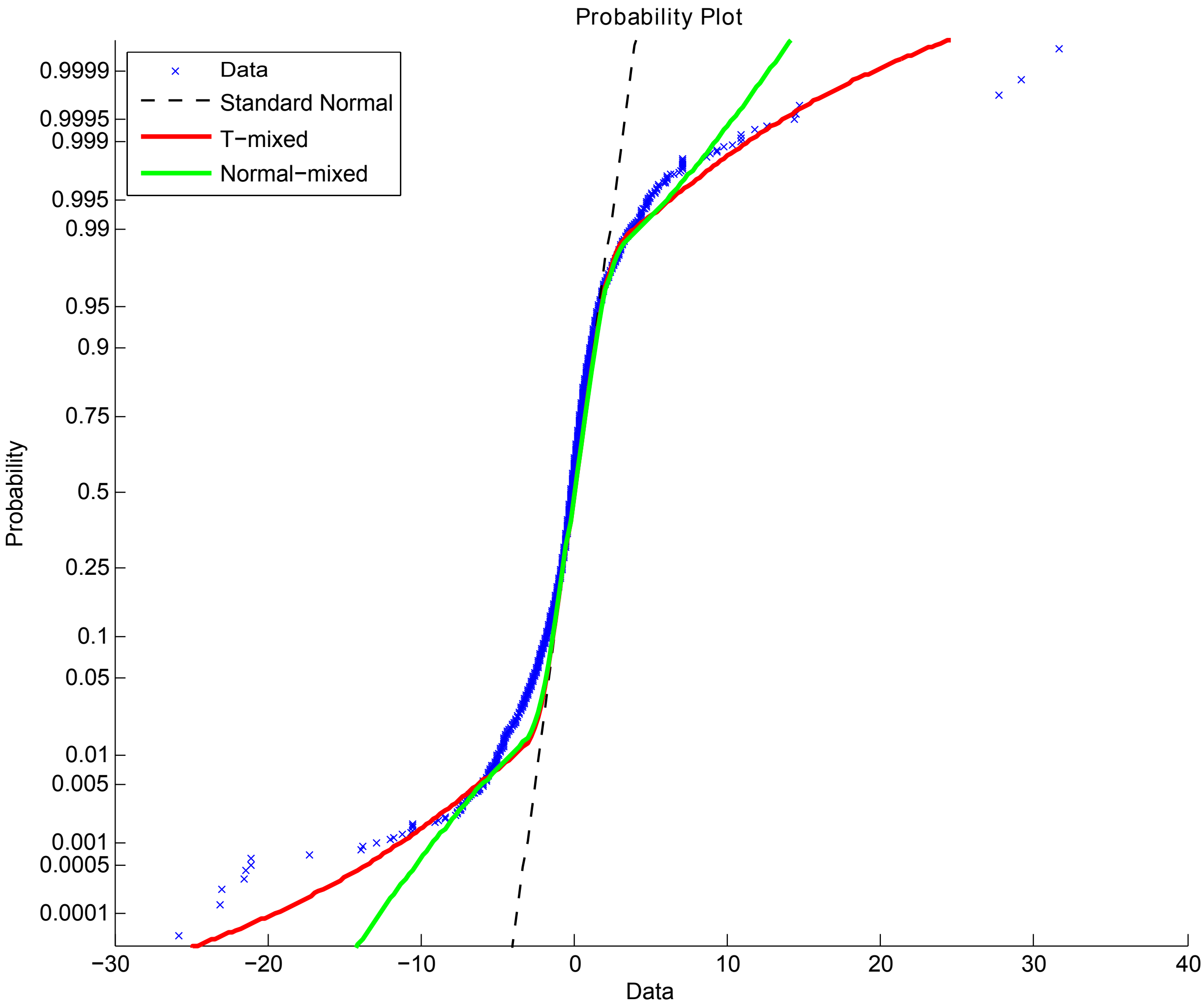

Supplementary figure 2: This figure shows how well different distributions fit to the sorafenib data. *Normal-mixed* is a mixture model of two normal distributions; *T-mixed* is a mixture of a normal and t location scale distribution.
